# Tuning Mg(OH)_2_ Structural, Physical, and Morphological Characteristics for Its Optimal Behavior in a Thermochemical Heat-Storage Application

**DOI:** 10.3390/ma14051091

**Published:** 2021-02-26

**Authors:** Elpida Piperopoulos, Marianna Fazio, Emanuela Mastronardo, Maurizio Lanza, Candida Milone

**Affiliations:** 1 Department of Engineering, University of Messina, 98166 Messina, Italy; faziom@unime.it (M.F.); cmilone@unime.it (C.M.); 2 National Interuniversity Consortium of Materials Science and Technology (INSTM), 50121 Florence, Italy; 3 Institute of Catalysis and Petrochemistry, Spanish National Research Council (CSIC), E-28049 Madrid, Spain; e.mastronardo@csic.es; 4 Institute for Chemical and Physical Processes (IPCF)—CNR, 98158 Messina, Italy; lanza@ipcf.cnr.it

**Keywords:** magnesium hydroxide, thermochemical energy storage, structural, physical and morphological characterization, stored and released heat

## Abstract

Thermochemical materials (TCM) are among the most promising systems to store high energy density for long-term energy storage. To be eligible as candidates, the materials have to fit many criteria such as complete reversibility of the reaction and cycling stability, high availability of the material at low cost, environmentally friendliness, and non-toxicity. Among the most promising TCM, the Mg(OH)_2_/MgO system appears worthy of attention for its properties in line with those required. In the last few decades, research focused its attention on the optimization of attractive hydroxide performance to achieve a better thermochemical response, however, often negatively affecting its energy density per unit of volume and therefore compromising its applicability on an industrial scale. In this study, pure Mg(OH)_2_ was developed using different synthesis procedures. Reverse deposition precipitation and deposition precipitation methods were used to obtain the investigated samples. By adding a cationic surfactant (cetyl trimethylammonium bromide), deposition precipitation Mg(OH)_2_ (CTAB-DP-MH) or changing the precipitating precursor (N-DP-MH), the structural, physical and morphological characteristics were tuned, and the results were compared with a commercial Mg(OH)_2_ sample. We identified a correlation between the TCM properties and the thermochemical behavior. In such a context, it was demonstrated that both CTAB-DP-MH and N-DP-MH improved the thermochemical performances of the storage medium concerning conversion (64 wt.% and 74 wt.% respectively) and stored and released heat (887 and 1041 $\mathrm{kJ}/{\mathrm{kg}}_{\mathrm{Mg}{\left(\mathrm{OH}\right)}_{2}}$). In particular, using the innovative technique not yet investigated for thermal energy storage (TES) materials, with NaOH as precipitating precursor, N-DP-MH reached the highest stored and released heat capacity per volume unit, ~684 MJ/m^3^.

## 1. Introduction

Thermal energy storage (TES) is a promising technology for leveling the demand peaks caused by the difference between energy production and consumption. A technology capable of managing demand peaks with greater efficiency, avoiding supply interruptions, and reducing the load where necessary is required [1,2,3]. In fact, in such a context, the research and development of the conversion and storage of waste heat are essential to overcome the energy mismatch between supply and demand. Furthermore, this contributes to decarbonization’s global purpose, to safeguard our planet’s health and contain disastrous climate changes [4,5]. Indeed, TES systems integrated into industrial processes and domestic users can face energy efficiency issues, improve the use of renewable resources, and reduce greenhouse gas emissions [6]. As one promising alternative to the storage of sensible [7,8] or latent heat [9,10,11], heat storage through reversible chemical reactions is under investigation [12,13]. By this method, the separated components of the thermochemical material (TCM) during the heating are stored in separate vessels to be recombined to generate heat when needed [14,15]. Therefore, this concept combines high process density and the capability to store energy as long as the reagents are far apart. Many reactions were proposed and analyzed, in this perspective [12,16]. Liu et al. studied redox pairs of Mn_2_O_3_/Mn_3_O_4_ and Co_3_O_4_/CoO [17], and Alonso et al. investigated the CuO/Cu_2_O system for solar energy thermochemical storage [18]. Furthermore, doped calcium manganites were recently proposed as TES materials, proposing a novel radiative model [19,20]. Among them, the Mg(OH)_2_/MgO system based on the reversible dehydration/hydration reaction:

Mg(OH)_2(s)_ ↔ MgO_(s)_ + H_2_O_(g)_     ∆H^0^ = ±81 kJ/mol (1)

has been extensively studied in the last few years [21,22] as originally proposed by Kato et al. [23]. Specifically, this material is promising for TES at medium temperature (300–400 °C) [24] because it demonstrates high theoretical stored heat (1389 J/g), being also inexpensive, abundant, non-toxic, non-flammable, and non-corrosive. These promising properties encouraged many researchers to study the optimization of the Mg(OH)_2_/MgO system for TES applications. Kurosawa et al. added LiCl and LiOH to Mg(OH)_2_, reaching a heat output density of 1053 kJ/kg [25]. Furthermore, Ishitobi et al. rose a heat output density per unit weight of LiCl-modified Mg(OH)_2_ of 1400 kJ/kg [26]. Shkatulov et al. doped magnesium hydroxide with sodium nitrate to tune the heat-storage material’s dehydration reactivity [27]. However, the real energy density is far from the theoretical one and generally decreases during the material’s cycling due to its deactivation, mostly caused by the hydroxide particles coalescence [28]. At this level, many research efforts were focused on increasing the material energy density. The effect of additives on the synthesis procedure was largely studied. Shkatulov et al. synthesized a new thermochemical (TC) composite material by precipitation of Mg(OH)_2_ in the pores of expanded vermiculite, reaching a heat storage capacity of 540 kJ/kg_composite_ with a hydroxide content of 67.4 wt.% [1]. Zamengo et al. used expanded graphite to increase heat-storage capacity up to 881 $\mathrm{kJ}/{\mathrm{kg}}_{\mathrm{Mg}{\left(\mathrm{OH}\right)}_{2}}$, enhancing at the same time its thermal conductivity [29]. Mastronardo et al. implemented Mg(OH)_2_ performance using exfoliated graphite and carbon nanotubes, obtaining hybrid materials raising the heat storage capacity up to 1000 $\mathrm{kJ}/{\mathrm{kg}}_{\mathrm{Mg}{\left(\mathrm{OH}\right)}_{2}}$ and 1300 $\mathrm{kJ}/{\mathrm{kg}}_{\mathrm{Mg}{\left(\mathrm{OH}\right)}_{2}}$, respectively [28,30]. Nevertheless, generally, the use of a support or secondary phase in the hybrid TCM preparation penalizes the energy density per volume unit, which is, among the others, one of the most relevant parameters for the engineering and applicability of the process. To address this issue, the research worked on mixed metal hydroxides, or doped magnesium hydroxide, which do not increase the volume of the inactive material but rather increase the TCM density to optimize the stored heat per volume unit. Shkatulov et al. recently worked on doped magnesium hydroxide, with the best results using LiNO_3_ doping to tune its thermogravimetric performance [31]. In our previous studies, we investigated the influence of metal doping in Mg(OH)_2_ synthesis, examining the thermochemical comportment, depending on different structural, morphological as well as thermochemical characteristics [32]. To determine if the thermochemical behavior of Mg(OH)_2_ may be improved without chemical doping, Müller and co-workers systematically studied the dehydration conditions’ effects on the subsequent rehydration reaction [33]. It was shown that dehydrated samples with larger Brunauer–Emmett–Teller (BET)-surface area resulted in the most reactive MgO-species, with higher re-hydration conversions [33]. Aiming to achieve high efficiency Mg(OH)_2_ synthesis production, Kim et al. increased the MgO surface area by forming micro-beams on the surface of the millimeter-size MgO pellets by electron beam irradiation [34]. On the other hand, we enhanced the heat storage aptitude per volume unit of Mg(OH)_2_ by adding the cationic surfactant CTAB (cetyl trimethylammonium bromide) during its synthesis, reaching, at an optimal CTAB concentration, the maximum volumetric stored and released heat capacity, ~560 MJ/m^3^ [35], associated to a significant increase of specific surface area and mean particle size reduction. Despite Mg(OH)_2_/MgO systems for TES application having been studied also in larger-scale reactors [36], the results achieved so far still have the potential to be improved for the applicability of this material at the industrial level. The use of alternative synthesis routes, not yet investigated, for the production of TES materials could be a way to achieve this goal. In such a context, considering what emerged from previous studies, this work aims to provide a correlation between the material’s structural, physical, and morphological characteristics (strictly determined by the synthesis route) and their thermochemical behavior. By comparing different synthesis methods and analysing the characteristics that follow, a close correspondence between the physical, morphological as structural characteristics of the examined material with its thermochemical performance is outlined. Within this scope, a correlation matrix was constructed through Pearson’s correlation coefficient, which gives a measure of the strength of a linear association between two random variables. A commercial Mg(OH)_2_ was also investigated for completeness. From this analysis a new approach is hypothesized for identifying the key parameters necessary for the recognition of the most performing material, in terms of conversion efficiency and storage capacity, thus providing guidance for tuning the thermochemical behaviour of other heat-storage materials. Indeed, by analysing the material’s stored energy density, its industrial application becomes feasible. In this context, the optimized material is identified and foundations are laid for all the researchers who work in the field of thermal energy storage materials to adopt an easier approach to fine-tune the acquired information in order to obtain a high-performance material. Additionally, as already mentioned above, enhancing the thermochemical performance of pure Mg(OH)_2_ is fundamental to increase the energy storage density. Indeed, the common approach of using a secondary phase (e.g., carbonaceous species), that does not participate in the heat storage/release reaction, sensibly reduces the heat storage capacity per mass or volume of total material that consists of both active and inactive components.

## 2. Experimental Section

### 2.1. Synthesis of Mg(OH)_2_

Commercial Mg(OH)_2_ was acquired by Kisuma Chemicals BV (98%, Veendam, The Netherlands). Synthesized samples were realized by different methods. The reverse deposition precipitation (RDP) method using NH_4_OH as precipitating agent was conducted following the reaction:

(2) $${\mathrm{Mg}(\mathrm{NO}}_{3}{)}_{2}{+2\mathrm{NH}}_{4}\mathrm{OH}\;\to {\;\mathrm{Mg}(\mathrm{OH})}_{2}{+2\mathrm{NH}}_{4}{\mathrm{NO}}_{3}$$

In detail, 150 mL of 1 M NH_4_OH (30 wt.%, Carlo Erba, Milan, Italy) were slowly added (2.5 mL/min) to 50 mL of 0.04 M Mg(NO_3_)_2_∙6H_2_O (99%, Sigma-Aldrich, Saint Louis, MO, USA) solution through a peristaltic pump during magnetic stirring (500 rpm), reaching a final pH of ~11.5, in agreement with literature [22].

A deposition precipitation (DP) method was compared with the previous one. In this specific case, the gradual addition of magnesium salt solution to the precipitating agent (pH ~11.5), under magnetic stirring, was performed [35].

Moreover, the cationic surfactant CTAB was added to the NH_4_OH solution in the percentage of 57 wt.% (final solution at pH of ~11.5), obtaining an additional sample [35].

The last precipitation method is an alternative technique not yet investigated for TES materials. It was achieved in the presence of a different precipitating agent, specifically 0.03 M NaOH (≥98%, Sigma-Aldrich, Saint Louis, MO, USA) solution (final pH of ~12.5) was used, following the reaction:

Mg(NO_3_)_2_ + 2NaOH → Mg(OH)_2_ + 2NaNO_3_ (3)

The final suspension was left at room temperature for 24 h, and subsequently, it was vacuum filtered (0.22 µm). The obtained solid was rinsed with deionized water and ethanol, and it was then vacuum dried at 60 °C overnight. Table 1 reports the samples’ codes and synthesis procedures.

### 2.2. Structural and Morphological Characterization of Mg(OH)_2_ Studied

The studied samples were examined employing X-ray diffraction (Bruker D8 Advance, Billerica, MA, USA) and Scanning Electron Microscopy (FEI Quanta 450, Hillsboro, OR, USA) to determine their crystal structure and their morphology. X-ray diffraction (XRD) was conducted in a 10–80° range, using a step of 0.1°/s, operating in Bragg-Brentano theta-2theta configuration, with Cu Kα radiation (40V, 40mA). Scanning electron microscopy (SEM) analysis was collected on Cr coated samples, with an accelerating voltage of 10 kV in high vacuum (10^−4^–10^−5^ Pa) [35].

Fourier-transform infrared spectroscopy (FTIR) was conducted using a Cary 670 Agilent spectrometer (Agilent Technologies, Santa Clara, CA, USA). The spectra were recorded from 500 to 4000cm^−1^ on KBr based pellets.

Surface area and pore volume were calculated by a Quantachrome Autosorb-iQ MP instrument (Anton Paar, Graz, Austria). examining nitrogen adsorption together with desorption isotherms (at −196 °C). Samples were first degassed under vacuum at the temperature of 120 °C and for 3 h. Specific surface area (S_BET_ (m^2^/g)) was quantified using the BET method, while pore volume (V_PORE_) was assessed by Barrett–Joyner–Halenda (BJH) approach on desorption isotherm [35].

Samples’ mean particle size was determined by dynamic light scattering (DLS) technique. DLS was performed at 25 °C using a Zetasizer Nano ZS instrument (Malvern Panalytical, Malvern, UK) equipped with a helium–neon 4-mW laser (wavelength λ_0_ = 632.8 nm). The scattering angle was equal to 173°. Samples were sonicated for 30 min in ethanol before measurement.

The samples bulk density (ρ) was quantified by weighing a specific volume of material (V (m^3^)) as explained by the Equation (4) (Figure S1 in Supplementary Materials):

(4) $$\rho \;=\;\frac{m\left(\mathrm{kg}\right)}{V\left(m^{3}\right)}$$

### 2.3. Thermochemical Performance of Mg(OH)_2_ Studied

The thermochemical attitude of the as-prepared samples under cyclic heat storage and release tests was studied using a customized thermogravimetric unit (STA 449 F3 Jupiter Netzsch) provided with a water vapor generator for the vapor provided during the hydration step [35].

The cyclic heat storage and release test was conducted as follows: the sample (~15 mg) was first dried at 125 °C in N_2_ atmosphere (100 mL/min) for 60 min to eliminate physically adsorbed water. Then, the temperature was raised (heating rate: 10 °C/min) up to the dehydration temperature (T_d_ = 350 °C), and it was maintained for 60 min, to perform the dehydration process. Following the whole dehydration step, the temperature was reduced (cooling rate = −10 °C/min) to the hydration temperature (T_h_ = 125 °C). The hydration reaction was conducted for 180 min, in a water vapor/N_2_ mixed flow (water vapor flow: 2.2 g/h, N_2_ flow: 35 mL/min as carrier gas). After the hydration step, the water vapor source was removed, and the sample was retained at 125 °C for 30 min in a constant inert flow (100 mL/min) to eliminate the physically adsorbed water. This approach is replicated for 3 heat storage/release cycles [35].

For consistency with prior studies [22,28,32,35,37], the examined samples were compared with regard to the reacted fraction (β(%)) defined by Equation (5):

(5) $$\beta \left(\%\right)\;=\;\left(1\;-\frac{\Delta m_{\mathrm{real}}}{\Delta m_{\mathrm{th}}}\right)\;\times \;100$$

where $\Delta m_{\mathrm{real}}\left(\%\right)$ was named the instantaneous real mass change and $\Delta m_{\mathrm{th}}\left(\%\right)$ was named the theoretical mass change owing to the dehydration of 1 mol Mg(OH)_2_, correspondingly indicated by Equations (6) and (7):

(6) $$\Delta m_{\mathrm{real}}(\%)\;=\;\frac{m_{\mathrm{in}}\;-{\;m}_{\mathrm{inst}}}{m_{\mathrm{in}}}\;\times \;100$$

(7) $$\Delta m_{\mathrm{th}}(\%)\;=\;\left(\frac{M_{\mathrm{Mg}{\left(\mathrm{OH}\right)}_{2}}\;-{\;M}_{\mathrm{MgO}}}{M_{\mathrm{Mg}{\left(\mathrm{OH}\right)}_{2}}}\;\times \;100\right)\;=\;30.89\%$$

where $m_{\mathrm{in}}\left(g\right)$ and $m_{\mathrm{inst}}\left(g\right)$ were correspondingly named the initial sample mass and the instantaneous mass in thermogravimetric (TG) analysis; whereas, $M_{\mathrm{Mg}{\left(\mathrm{OH}\right)}_{2}}\left(g/\mathrm{mol}\right)$ and $M_{\mathrm{MgO}}\left(g/\mathrm{mol}\right)$ were named correspondingly the molecular weight of Mg(OH)_2_ and MgO.

The dehydration in addition to the hydration conversions ($\Delta \beta_{d/h}\left(\%\right)$) were calculated, correspondingly, by Equations (8) and (9):

(8) $$\Delta \beta_{d}{(\%)\;=\;\beta }_{d}^{i}\;-\beta_{d}^{f}$$

(9) $$\Delta \beta_{h}{(\%)\;=\;\beta }_{h}-\beta_{d}^{f}$$

where $\beta_{d}^{i}$ and $\beta_{d}^{f}$ were correspondingly the reacted fraction at the start point and at the end of the dehydration step, while, $\beta_{h}$ was the final reacted fraction of MgO at the end of the water supply.

The stored and released heat per mass ($Q_{s/r}^{M}(\mathrm{kJ}/{\mathrm{kg}}_{\mathrm{Mg}{\left(\mathrm{OH}\right)}_{2}}$)) and volume unit ($Q_{s/r}^{V}{(\mathrm{MJ}/m}^{3})$) were calculated respectively by Equations (10) and (11):

(10) $$Q_{s/r}^{M}(\mathrm{kJ}/{\mathrm{kg}}_{\mathrm{Mg}{\left(\mathrm{OH}\right)}_{2}})\;=\;-\;\frac{\Delta H^{0}}{M_{\mathrm{Mg}{\left(\mathrm{OH}\right)}_{2}}}\;\times \;\Delta \beta_{d/h}$$

(11) $$Q_{s/r}^{V}{(\mathrm{MJ}/m}^{3})\;=\;-\;\frac{\Delta H^{0}}{M_{\mathrm{Mg}{\left(\mathrm{OH}\right)}_{2}}}\;\times \;\Delta \beta_{d/h}\;\times \;\rho$$

where ∆H^0^ (kJ/mol) is the reaction enthalpy, and ρ (kg/m^3^) the bulk density of the sample.

## 3. Results and Discussion

### 3.1. Structural, Morphological and Physical Characterization of the Samples Studied

**Figure 1:**
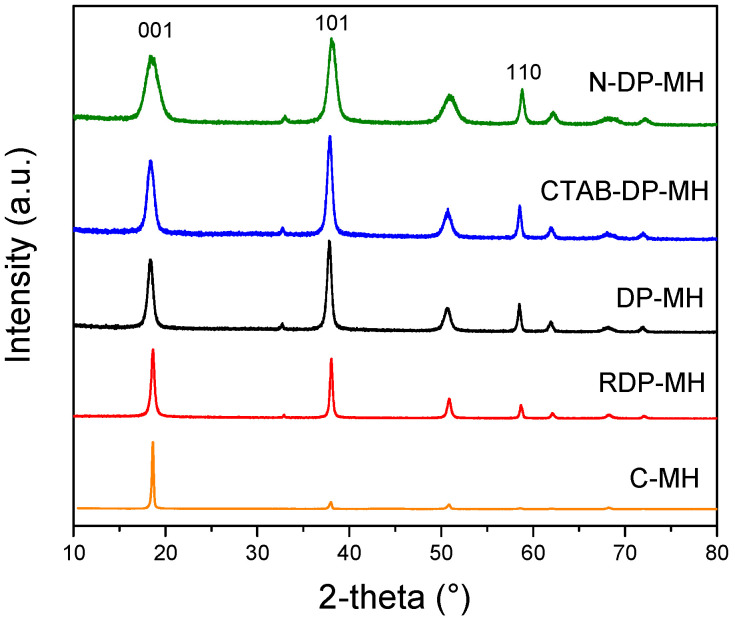
X-ray diffraction (XRD) analysis of investigated samples.

XRD analysis was conducted in the 2-theta range 10°–80°. Diffractograms show brucite phase (JCPDS 7-0239) for all the samples examined, in accordance with peaks positions and intensities. No impurities were distinguished. Characteristic XRD diffractograms normalized with regard to the [001] peak is presented in Figure 1. Comparing the three brucite characteristic peaks (2-theta = 18.5°, 38.0°, 58.5°) for the inspected samples, the C-MH pattern is totally different from the synthesized samples. The [001] peak looks strongly sharp and narrow. Furthermore, all the other peaks, considerably smaller than the [001] one, are hidden at the reported graph scale (e.g., peaks at 33° and 62°). The peaks of RDP-MH appear sharper and narrower than those obtained by other synthesis conditions as a result of the higher degree of crystallization of brucite. DP-MH and CTAB-DP-MH exhibit very similar patterns and peaks’ normalized intensities, while less intense and broader peaks are observable for N-DP-MH. Generally, the peaks broadening in powder XRD patterns of polycrystalline solids are attributed to particle size effects [38]. The wider the peak, smaller the particle size.

Table 2 lists the intensity ratio of reflections [001], [101], and [110] for the samples and their physical properties. The highest I001/I101 and I001/I110 ratios are reported for the C-MH sample, whose ratios reach 10.1 and 93.32, respectively, confirming the clear difference between the peak at 18.5° and the other two brucite characteristic peaks at 38.0° and 58.5°. Although lower compared to C-MH, the RDP-MH sample presents higher intensity ratios than the samples obtained by the DP method, corroborating the thesis that the different synthesis method structurally influences the obtained materials [21]. As reported elsewhere [39], the higher intensity of [001] plane is representative of layered structures, e.g., flakes or platelets, designated with an elevated aspect ratio onwards the c axis and a compact morphology.

XRD intensity ratios of [001] with respect to [101] and [110] reflections (I001/I101, I001/I110) of DP-MH sample do not significantly vary compared to the other samples obtained by DP synthesis method (CTAB-DP-MH and N-DP-MH), suggesting that no significant differences in the growth rate of different crystallographic planes occur when CTAB is present or using NaOH as a precipitating agent. In particular, in the latter case, a decrease in I001/I110 ratio is evidenced. From the intensities of the [001] and [110] reflections, it can be possible to get an indication of the particles’ orientation. Usually, in a hexagonal symmetry system, the common direction of lamellar particles is [001], as confirmed by RDP-MH sample, which I001/I110 ratio is the highest. The [001] diffraction of DP-MH and CTAB-DP-MH (2.52 and 2.27, respectively) samples is more intense than that of the N-DP-MH sample (1.90), indicating a more pronounced orientation of the hydroxide particles towards the incident X-ray radiation [39].

**Figure 2:**
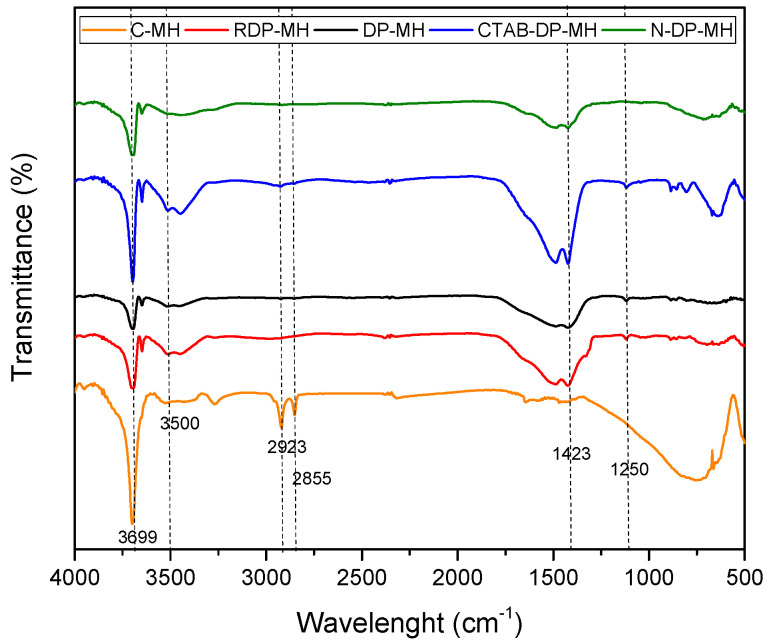
Fourier transform infrared (FT-IR) analysis of investigated samples.

The structure of Mg(OH)_2_ was confirmed by FTIR spectroscopy for all the samples (Figure 2). In the spectrum, the sharpest and most intense peak at 3699 cm^−1^ is assigned to the OH antisymmetric stretching vibration. Its intensity significantly differs from one sample to another. Generally, an increase in the peak intensity usually means an increase in the amount per unit volume of the functional group associated with the molecular bond. The peaks from 500 to 1250 cm^−1^ account for the metal hydroxide stretching vibrations. The OH stretching vibration appeared around 3500 cm^−1^. The broad peak at 1423 cm^−1^ could be associated to the bending vibration of OH bond. Only the commercial MH, C-MH sample, exhibits two extra peaks at 2923 and 2855 cm^−1^, associated with a saturated C–H vibration, related to stabilizers or minor impurities [40,41].

**Figure 3:**
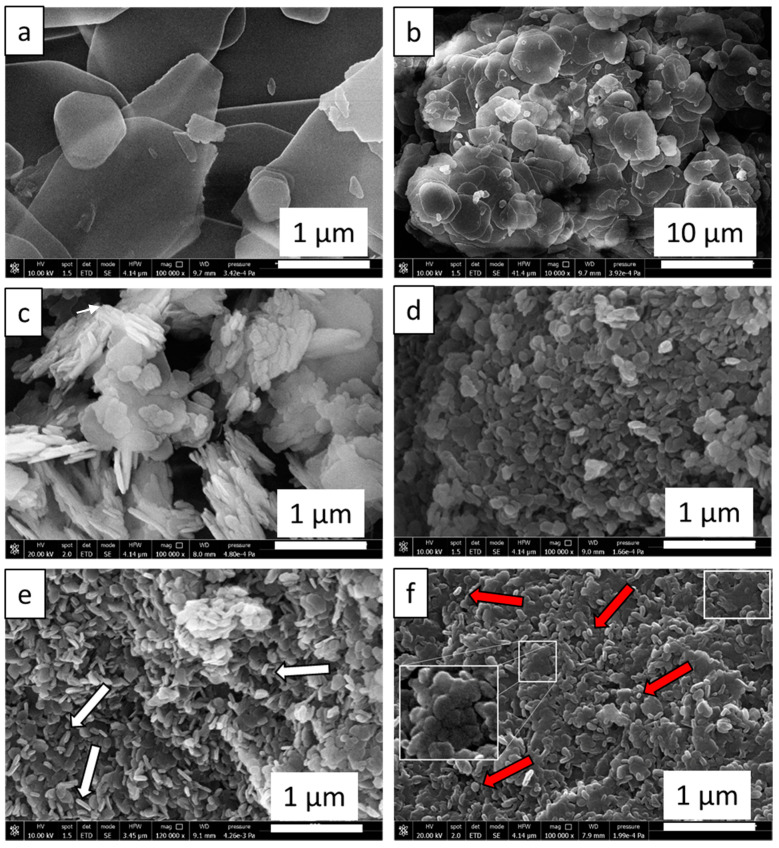
Scanning electron microscope (SEM) images of (**a**) C-MH, (**b**) C-MH at low magnification, (**c**) RDP-MH, (**d**) DP-MH, (**e**) CTAB-DP-MH, (**f**) N-DP-MH samples.

From SEM analysis, shown in Figure 3, C-MH is composed of thin Mg(OH)_2_ plates with an irregular shape (Figure 3a) notably larger than all the other samples, corroborating the XRD pattern in Figure 1. These particles tend to aggregate forming bulky agglomerations (see Figure 3b). For RDP-MH (Figure 3c), we can note the presence of large not homogeneous platelet aggregates. Using the deposition precipitation method, the platelets appear hexagonal and smaller than the previous ones (Figure 3d). The CTAB addition leads to hydroxide particles mostly distinct in separate grains with a recognizable contour and a needle-like morphology (see white arrows in Figure 3e). However, as previously reported [35], acquiring the image, tilting the sample holder by 45°, confirms a hexagonal grain shape. The observed needle-like shape is caused by the predominant orientation of [101] brucite faces towards the electron beam, probably due to the particles’ particular stacked configuration. N-DP-MH sample appears similar to CTAB-DP-MH, but some globular magnesium hydroxide particles (see red arrows in Figure 2f) are detected, and a more packed morphology is visible (white rectangles in Figure 3f). The image acquisition at higher magnification confirms that agglomerations are formed by small hexagonal particles with well-defined contours (see projection in Figure 3f).

**Figure 4:**
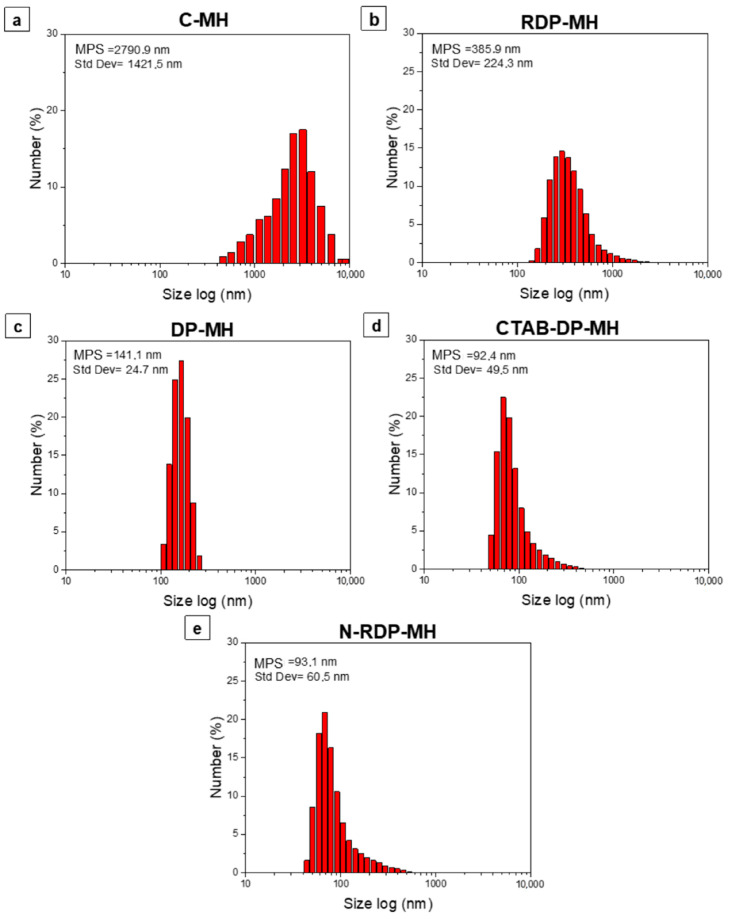
Dynamic light scattering (DLS) size distribution by number (%) of (**a**) C-MH, (**b**) RDP-MH, (**c**) DP-MH, (**d**) CTAB-DP-MH, (**e**) N-DP-MH.

DLS analysis of the investigated samples are represented in Figure 4. The mean particle size (MPS) and standard deviation are recorded for all the samples. The particle size distribution of the C-MH sample is the only one shifted to values higher than 1000 nm, up to over 5000 nm (Figure 4a). The sample, as indicated in SEM analysis (Figure 3a) and confirmed by XRD pattern in Figure 1, is formed by large particles with a mean size of ≈2791 nm, agglomerated in larger and bulky clusters (Figure 3b). As inferred from the SEM images, the RDP-MH sample is made of Mg(OH)_2_ particles with plate-like morphology and an average size of ≈120 nm, which tend to aggregate, forming bigger flakes of ≈386 nm. It is observed that the effect of the synthesis procedure is a lowering of the average particle size, from 386 nm (Figure 4b) for RDP-MH up to 141 nm (Figure 4c) for DP-MH. A further decrease in the mean diameter of the hydroxide particles is achieved by adding CTAB in the synthesis procedure or changing the precipitating agent with NaOH, as is visible in Figure 4d,e. The smaller particle size, 92 nm and 93 nm, respectively for CTAB-DP-MH and N-DP-MH, is accompanied by a broadening of size distribution compared to the DP-MH sample.

Additionally, Table 2 lists the physical features of the considered samples, such as specific surface area (S_BET_ (m^2^/g)), pore volume (V_PORE_ (cm^3^/g)), and bulk density (ρ (kg/m^3^)). From the aforementioned data, the surface area of C-MH results in the lowest, 7.0 m^2^/g, accompanied by the lowest pore volume (0.020 cm^3^/g) and a density of 405 kg/m^3^ confirming the SEM images and DLS analysis. Conversely, it is clear that the deposition precipitation method allows the DP-MH to show higher surface area (64.5 m^2^/g) and pore volume (0.618 cm^3^/g) in comparison with RDP-MH sample (44.4 m^2^/g and 0.112 cm^3^/g respectively), while maintaining almost the same density (~345 kg/m^3^), that is lower than the commercial sample. In such a context, a considerable increase in density is recorded for the CTAB-DP-MH and N-DP-MH samples (602 kg/m^3^ and 660 kg/m^3^, respectively), accompanied by an increase in pore volume (0.735 cm^3^/g and 0.723 cm^3^/g) and an increase in the surface area (85.6 m^2^/g for CTAB-DP-MH), especially in the case of the N-DP-MH sample (124.5 m^2^/g).

This similarity between the two samples is made even more reliable by the DLS analysis (Figure 4) of the two materials, which report almost the same size of particles, and by the peculiar morphology found in the SEM analysis (Figure 3).

**Figure 5:**
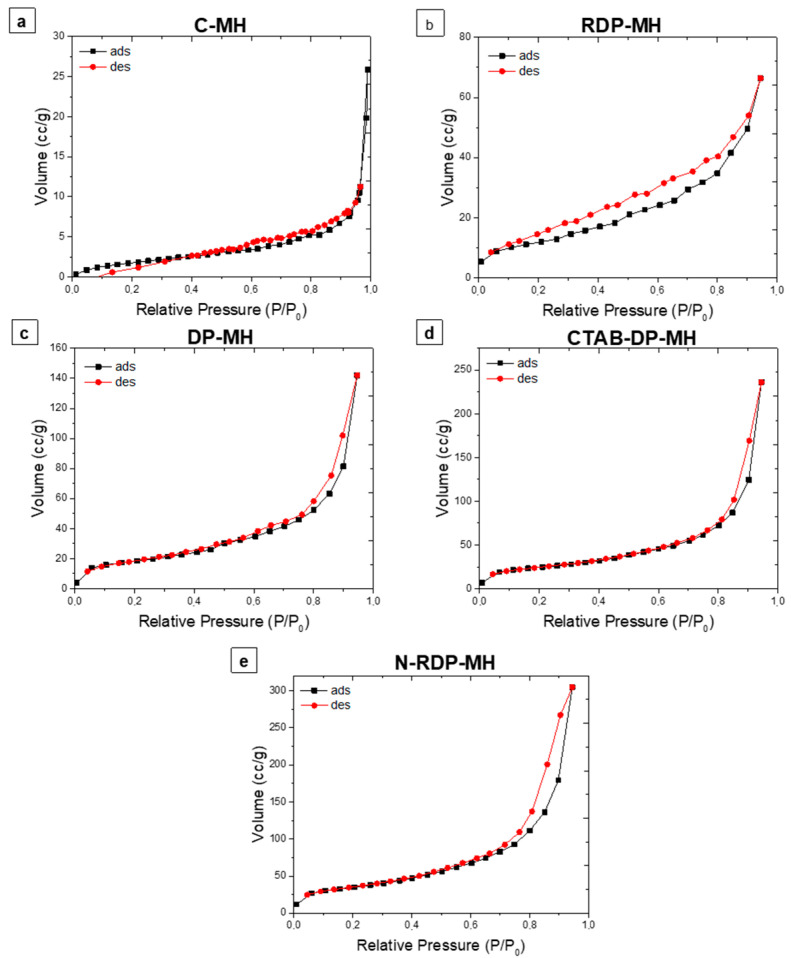
Isothermal physisorption curves of (**a**) C-MH, (**b**) RDP-MH, (**c**) DP-MH, (**d**) CTAB-DP-MH, (**e**) N-DP-MH.

The isothermal physisorption curves of the studied samples are reported in Figure 5. All samples show type IV isothermal curves that are characteristic of mesoporous materials [42]. The C-MH isotherm does not exhibit hysteresis loops, maybe for the very low surface area and pore volume of the sample. The RDP-MH isotherm shows a type H-4 loop, nearly horizontal and parallel over a wide range of P/P_0_. This type of hysteresis is often associated with narrow slit-like pores. For DP samples, the desorption curve comprehends a steep region associated with a conclusion of the hysteresis loop in the P/P_0_ range from 0.4–0.45, owing to the tensile strength effect. Therefore, as stated by IUPAC classification [42], the isotherms are designated by a type H-3 hysteresis that does not show any restrictive adsorption at high P/P_0,_ and it occurs with agglomerations of plate-like particles inducing slit-shaped pores.

**Figure 6:**
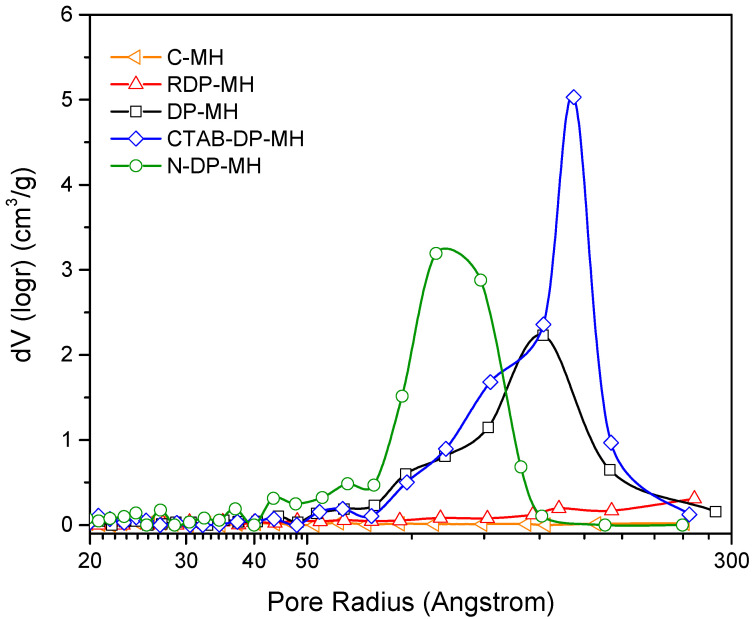
Volume of the pores in relation to their average size for each investigated sample.

The notably larger particles sizes of C-MH and RDP-MH with respect to the rest of the samples explains their significantly smaller specific surface area and pore volume (especially in C-MH). As shown in Table 2, pore volume (V_PORE_) does not significantly differ among DP-MH, CTAB-DP-MH, and N-DP-MH samples; a small increase by 18% is found from DP-MH to CTAB-DP-MH and N-DP-MH samples. By contrast, the surface area of these samples varies considerably. Comparing the DP-MH sample and the CTAB-DP-MH (see Table 2), the increase in surface area can be associated with the samples’ specific morphological shape and size (Figure 3). Indeed, as mentioned above, with respect to DP-MH, in CTAB-DP-MH smaller and well separated single particles with distinguishable contours are observed. What does not seem to be explained is the increase in the N-DP-MH surface area compared to the CTAB-DP-MH, with which it shares almost the same morphological aspect and particle size. This can be understood in light of the results shown in Figure 6, which report the pores’ volume in relation to their average size.

As can be discerned, the N-DP-MH sample, while maintaining almost the same total pore volume as the CTAB-DP-MH sample (Table 2), has a smaller pore size, thus creating a more packed structure, as evidenced in SEM analysis (Figure 3f), explaining the higher material’s density and higher surface area, considering the clearly distinguishable and separate hydroxide particles.

#### Remark on the Role of the Synthesis Route on Mg(OH)_2_ Structural, Physical, and Morphological Characteristics

From the characterization outcomes exhibited so far, it can be believed that the synthesis procedure does not generate any structural modification of the hydroxide, as deduced by XRD analysis, but it strictly influences morphological (S_BET_, MPS, V_PORE_) and physical characteristics (bulk density) of the produced samples. In particular, as demonstrated by previous studies [22], RDP and DP supersaturation degree has a different increasing rate during the synthesis procedure. This relentlessly influences the morphology of the synthesized samples. In such a context, under the condition employed in the present study, in which the [Mg^2+^] concentration in the final solution is 0.01 M, the supersaturation degree is reached at pH = 9.5 and increases with the pH increase. Thus, in RDP the supersaturation degree slowly increases from 7.5 up to 11.5, and this slow rise brings the formation of few crystals, which are the seeds for additional nucleation and crystallization. Therefore, bigger particles form, entailing smaller surface area, pore volume, and density. Conversely, by the DP method, the hydroxide precipitation happens at constant pH = 11.5 and the fast achievement of the high supersaturation degree endorses the formation of a greater number of smaller crystals. Consequently, larger surface area, pore volume, and density are achieved with respect to the RDP-MH sample. The presence of CTAB cationic surfactant in the form of micelles favors the development of very distinct single grains because the high amount of CTAB micelles, with a positively charged surface, present in solution act as nucleation seeds for the Mg(OH${)}_{6}^{4-}$ basic unit that is negatively charged. Mg(OH${)}_{6}^{4-}$ promotes the Mg(OH)_2_ crystals formation, piling up, and growth [35]. An increase in surface area, pore volume, and density is observed with respect to DP-MH sample. Furthermore, NaOH as precipitating agent promotes the formation of well-separated Mg(OH)_2_ particles with higher S_BET_ and smaller mean particle size, which contributes to a more dense structure. It can be postulated that the pH of the solution and the ions’ nature act as a critical factor during crystal growth [43,44]. The isoelectric point of the magnesium hydroxide particles in water is determined at pH 12. Concerning the synthesis with NaOH employed as precipitating agent, the pH attained in the reacting mixture throughout the synthesis is the highest (~12.5). Therefore, the elevated supersaturation mentioned before is also accompanied by a negative electric charge on the hydroxide particles’ surface. From this, an extraordinarily rapid nucleation process derives, forming minuscule nuclei. Succeeding the nucleation phase, cations inside the solution move en route for the developing crystals. Because of the high concentration and the small dimension of sodium ions, they can adsorb considerably and indiscriminately on the nuclei faces, not allowing the new magnesium ions access and the following growth [45]. These small and isotropic units favor their aggregation to decrease the surface energy, finally assuming the dense structure reported in Figure 3f and in agreement with the observed smaller pore size (Figure 6).

### 3.2. Thermochemical Performance of Mg(OH)_2_ Samples

**Figure 7:**
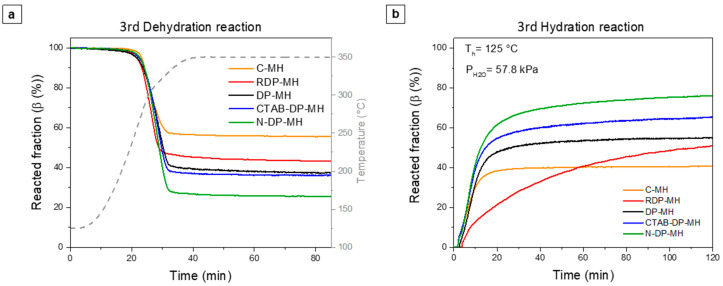
(**a**) Dehydration and (**b**) hydration curves at the 3rd cycle.

The thermochemical comportment of Mg(OH)_2_ samples was investigated by three dehydration and hydration cycles tests under settings reported in Section 2.3. Figure 7 displays the dehydration and hydration step curves related to the third cycle, when the thermochemical comportment of the samples is considered stable [28,30]. The figure shows the reacted fraction (β (%)) vs. reaction time (t (min)). 

As shown by Figure 7, all the dehydration curves end with a plateau, and after almost 50 min, the reaction can be judged terminated. The hydration curves show different behavior for the examined samples. C-MH concludes the hydration process after 30 min, settling at the lowest level of hydration. The RDP-MH sample shows completely different kinetics with respect to the other samples, not reaching complete hydration even after 120 min.

Table 3 reports the dehydration/hydration conversion (Δβ_d/h_), and the stored and released heat capacity per mass unit ($Q_{s/r}^{M}$) and volume unit ($Q_{s/r}^{V}$) at the third cycle, for better comprehension.

The lowest dehydration and hydration conversions of the thermochemical material are recorded for the commercial product (44.4% and 40.7%, for dehydration and hydration, respectively). There is a clear difference in conversion using deposition precipitation as a magnesium hydroxide synthesis procedure. In fact, the efficiency increases in the latter case (62.8% vs. 56.7% in dehydration and 55.5% vs. 50.9% in hydration, respectively, for DP-MH and RDP-MH). 

**Figure 8:**
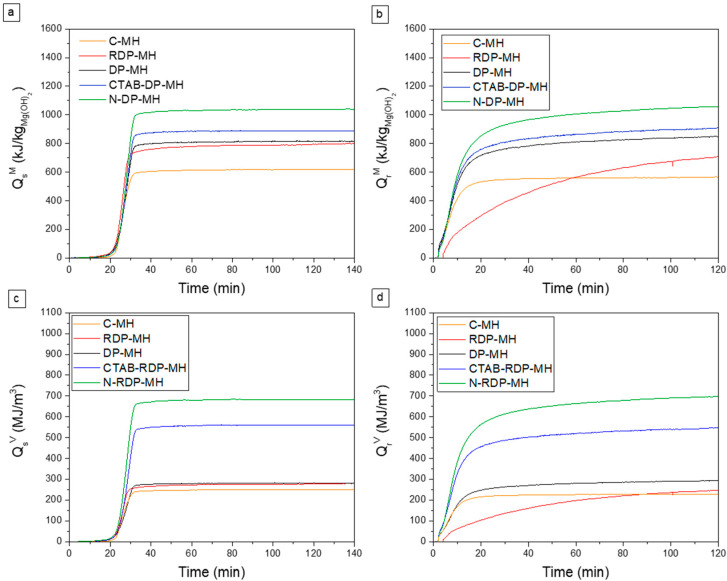
(**a**,**c**) Stored and (**b**,**d**) released heat per mass unit and volume unit at the 3rd cycle.

It is noteworthy that only in the case of CTAB-DP-MH and N-DP-MH samples the hydration conversion (Δβ_h_) nearly equals the dehydration conversion values (Δβ_d_), consequently indicating the almost total transformation of earlier dehydrated MgO in Mg(OH)_2_. Conversely, the reaction reversibility is not respected for the other samples, probably due to unreacted MgO [35]. Furthermore, N-DP-MH shows the highest dehydration/hydration conversion (~76%), the highest value found so far in the literature for pure MH. Observing the massive stored and released heat capacity ($Q_{s/r}^{M}$) (Figure 8a and b), it can be noticed that C-MH reveals the lowest stored and released heat capacity that is 617 $\mathrm{kJ}/{\mathrm{kg}}_{\mathrm{Mg}{\left(\mathrm{OH}\right)}_{2}}$ and 565 $\mathrm{kJ}/{\mathrm{kg}}_{\mathrm{Mg}{\left(\mathrm{OH}\right)}_{2}}$ (Table 3). In line with the trend of just described conversion efficiency, the highest value is recorded for the N-DP-MH sample, which reports a stored heat of 1041 $\mathrm{kJ}/{\mathrm{kg}}_{\mathrm{Mg}{\left(\mathrm{OH}\right)}_{2}}$ and a released heat of 1056 $\mathrm{kJ}/{\mathrm{kg}}_{\mathrm{Mg}{\left(\mathrm{OH}\right)}_{2}}$.

With regard to the volumetric stored and released heat ($Q_{s/r}^{V}$ in Figure 8c,d), C-MH exhibits the lowest values (~250 MJ/m^3^). Furthermore, no noticeable differences are detected between the sample obtained by RDP (302 MJ/m^3^) and that by DP (~280 MJ/m^3^), while there is a clear improvement when DP is carried out with CTAB surfactant (~560 MJ/m^3^) or in NaOH medium, as a reagent. N-DP-MH is also the sample with the highest volumetric stored and released heat (~690 MJ/m^3^). This result is easily understandable, looking at Table 2, where the material’s density is reported as the highest among the considered samples. With respect to the authors’ previous study, where a heat storage density of 640.75 MJ/m^3^ is achieved with co-doped MH, N-DP-MH exhibits larger heat storage capacity. Moreover, while many studies do not report the material density, it is fundamental for a component design perspective, with respect to other materials in which a secondary phase is present, and a larger heat storage density is clearly expected due to the lack of an inactive component.

**Figure 9:**
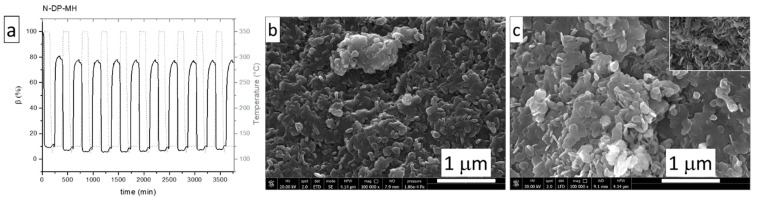
N-DP-MH thermochemical stability over ten cycles experiment (**a**); SEM images of as-prepared N-DP-MH (**b**) and of N-DP-MH after 10-cycle experiment (**c**).

In light of its favorable thermodynamic properties and heat storage density, N-DP-MH emerges as an attractive candidate for thermochemical heat storage applications. Accordingly, it was further investigated in terms of cyclability over multiple dehydration/re-hydration reactions. The mass profiles collected over 10 cycles are plotted in Figure 9a, where the dehydration was carried out upon heating at 10 °C/min to 350 °C under inert gas (N_2_ 100 mL/min) and the subsequent re-hydration at 125 °C under *p*_H2O_ of 57.8 kPa. Except for the first cycle, the material exhibits a stable reaction conversion (β (%)) of about 76% over all the duration of the experiment. Thus, N-DP-MH appears suitable for long life-time heat storage.

SEM analysis of the N-DP-MH sample after a ten-cycle experiment is reported in Figure 9b. Despite a slight coalescence in comparison with the as-prepared material (Figure 9a), the morphology of the sample results almost unmodified. The hydroxide particles, as shown in the reported image magnification, appear clearly distinguishable and the contours well defined.

### 3.3. Cross-Correlation between Mg(OH)_2_ Physical, Morphological and Thermochemical Variables

**Figure 10:**
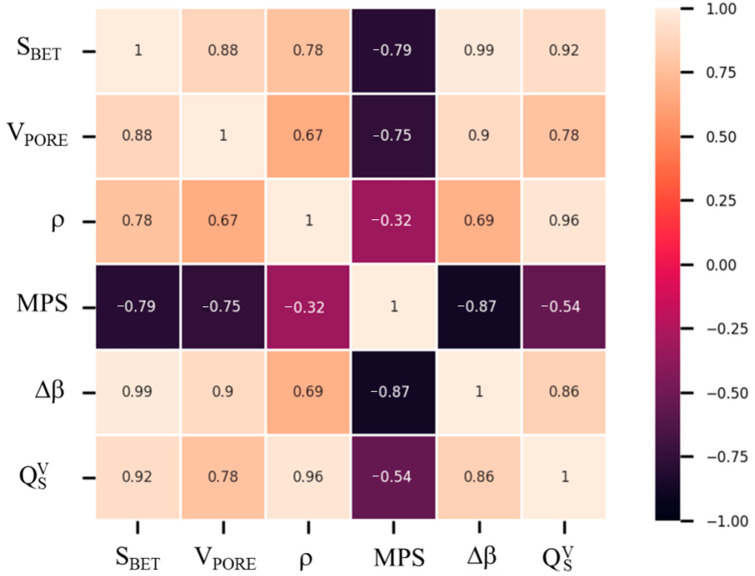
Correlation matrix of samples’ physical, morphological and thermochemical characteristics.

The main results achieved are strictly dependent on the structural, physical, and morphological characteristics of the obtained samples. In Figure 10, a correlation matrix showing the correlation coefficients between the investigated physical, morphological and thermochemical variables is reported. The correlation matrix was constructed calculating the Pearson’s correlation coefficient, which is a measure of the strength of a linear association between two random variables and denoted by *r*. Given a number of measurements *n* of the (X, Y) pair variables, the *r* coefficient was calculated by the following Equation (12) [46,47]:

(12) $$r_{xy}\;\overset{def}{=}\;\frac{\sum_{i=1}^{n}\left(x_{i}-\bar{x}\right)(y_{i}-\bar{y})}{\left(n-1\right)s_{x}s_{y}}\;=\;\frac{\sum_{i=1}^{n}\left(x_{i}-\bar{x}\right)\left(y_{i}-\bar{y}\right)}{\sqrt{\sum_{i=1}^{n}{\left(x_{i}-\bar{x}\right)}^{2}\sum_{i=1}^{n}{\left(y_{i}-\bar{y}\right)}^{2}}}$$

where $r_{xy}$ is the Pearson’s correlation coefficient for the generic variables X and Y. $x_{i}$ and $y_{i}$ are the *i*-th variables X and Y, $\bar{x}$ and $\bar{y}$ are the mean values and $s_{x}$ and $s_{y}$ are the corrected sample standard deviations of X and Y. $r_{xy}$ has a value between +1 and −1, which are, respectively, total positive and negative linear correlation, 0 is where no correlation is detected. The resulting matrix is a 6 × 6 square where 6 is the number of considered samples’ characteristics:

1. specific surface area (S_BET_);
2. pore volume (V_PORE_);
3. density (ρ);
4. mean particle size (MPS);
5. reacted fraction (Δβ);
6. volumetric heat storage capacity ($Q_{S}^{V}$).

Each matrix coefficient is calculated based on Equation (12). Figure 10 shows that a correlation exists between the investigated variables. In fact, almost all the coefficients reported in the matrix have a value above 0.50. Only in the case of the density ρ and the mean particle size MPS variables is a lower value (0.34) recorded. Furthermore, for the pairs of variables (S_BET_, Δβ and (S_BET_, $Q_{S}^{V}$), almost 1 is reached, confirming a linear correspondence between the surface area and the material’s conversion, also intended as heat stored per volume unit. A further parameter connected to the surface area and related to the material’s conversion is the pore volume (V_PORE_), whose related coefficients are 0.88 and 0.90, respectively. Additionally, a close relationship exists between the density value of the thermochemical material and the stored heat per volume unit, as can be easily deduced. Therefore, it follows that a material with a high surface area, a high pore volume, and a high density could guarantee an optimal conversion and a consequent high stored heat per volume unit. In such a context, the N-DP-MH sample is taken into consideration. N-DP-MH, whose high S_BET_ (124.5 m^2^/g), as previously reported, depends on the size of the clearly distinguishable particles (93 nm), confirmed by SEM images and by the high pore volume (0.723 cm^3^/g), and reports higher conversion efficiency (75%).

Moreover, N-DP-MH has higher density (660 kg/m^3^) due to the particular morphology formed by globularly shaped hydroxide particles. This morphological characteristic allows the sample to reach a stored heat per volume unit equal to 684 MJ/m^3^. This value is one of the highest recorded in the literature for pure magnesium hydroxide and represents a promising result toward the aim of thermochemical energy storage. Furthermore, the previously described approach can help to more easily identify the parameters influencing the requested performances of TCM and to tune the synthesis towards the optimal product.

## 4. Conclusions

The outcomes of the presented study propose that Mg(OH)_2_ synthesis route strictly influences the physical (bulk density) and morphological (S_BET_) features of the achieved samples. Specifically, it has been established that the reverse deposition precipitation method conducts an agglomerated material composed of irregular plate-like morphology particles that exhibit less surface area and less pore volume (V_PORE_). Conversely, by the deposition precipitation method, the synthesized sample presents an increase in S_BET_ and in V_PORE_, due to the presence of smaller hydroxide hexagonal plates. In such a context, this result is further improved using CTAB surfactant in the course of the synthesis procedure or substituting NH_4_OH by NaOH as precipitating agent. In fact, in these cases, the samples obtained show a higher S_BET_ and a particular configuration, in which Mg(OH)_2_ particles are clearly distinguishable. In particular, the N-DP-MH sample presents a surface area of 124.5 m^2^/g, correlated to the small particle size and a peculiar configuration of globular particles, contributing to the density increase (660 kg/m^3^).

A correlation between physical and morphological characteristics and the thermochemical behavior of Mg(OH)_2_ is found, using a correlation matrix as a tool. This approach can be useful to identify the influencing parameters in TCM performances and to optimize research results. In particular, the higher the S_BET_ and the bulk density, the higher the efficiency conversion (Δβ) and thus the storage-released heat per volume unit ($Q_{s/r}^{V}$).

As the main result, N-DP-MH indicates the highest massive stored and released heat capacity, ~1040 $\mathrm{kJ}/{\mathrm{kg}}_{\mathrm{Mg}{\left(\mathrm{OH}\right)}_{2}}$ so far mentioned in literature, and the highest heat capacity per volume unit (~690 MJ/m^3^). This makes the sample promising for thermochemical energy storage applications.

## Figures and Tables

**Table 1:** Samples’ codes, synthesis procedures.

Code	Synthesis Procedures
C-MH	Commercial Mg(OH)_2_
RDP-MH	Reverse deposition precipitation
DP-MH	Deposition precipitation
CTAB-DP-MH	Deposition precipitation in presence of CTAB
N-DP-MH	Deposition precipitation using NaOH as precipitating agent

**Table 2:** Structural and morphological properties of the investigated samples.

Code	Intensity Ratios		Physical Properties		
	I_001_/I_101_	I_001_/I_110_	S_BET_ (m^2^/g)	V_PORE_ (cm^3^/g)	ρ (kg/m^3^)
C-MH	10.1	93.32	7.0	0.020	405 ± 2
RDP-MH	1.1	5.70	44.4	0.112	350 ± 5
DP-MH	0.8	2.52	64.5	0.618	345 ± 6
CTAB-DP-MH	0.8	2.27	85.6	0.735	602 ± 6
N-DP-MH	0.8	1.90	124.5	0.723	660 ± 4

**Table 3:** Dehydration/hydration conversions (Δβ_d/h_) and stored and released heat per mass unit ($Q_{s/r}^{M}$) and volume unit ($Q_{s/r}^{V}$) at the third cycle.

Code			3rd Cycle			
	Δβ_d_ (%)	Δβ_h_ (%)	$Q_{s}^{M}$ $\left(\mathrm{kJ}/{\mathrm{kg}}_{\mathrm{Mg}{\left(\mathrm{OH}\right)}_{2}}\right)$	$Q_{r}^{M}$ $\left(\mathrm{kJ}/{\mathrm{kg}}_{\mathrm{Mg}{\left(\mathrm{OH}\right)}_{2}}\right)$	$Q_{S}^{V}$ (MJ/m^3^)	$Q_{r}^{V}$ (MJ/m^3^)
C-MH	44.4 ± 1.3	40.7 ± 1.5	617 ± 7.5	565 ± 7.7	250 ± 11.0	230 ± 10.8
RDP-MH	56.7 ± 1.5	50.9 ± 1.6	797 ± 9.8	707 ± 8.6	280 ± 12.0	248 ± 11.6
DP-MH	62.8 ± 1.6	55.5 ± 1.6	872.9 ± 9.6	771.4 ± 8.8	302 ± 7.5	254 ± 6.3
CTAB-DP-MH	66.8 ± 1.5	67.0 ± 1.5	928.5 ± 11.0	931.2 ± 10.3	559 ± 13.9	561 ± 14.0
N-DP-MH	74.6 ± 1.6	76.1 ± 1.6	1041 ± 10.2	1056 ± 9.5	684 ± 11.3	698 ± 12.3

## Data Availability

Data is contained within the article or Supplementary Materials.

## Supplementary Materials

The following are available online at https://www.mdpi.com/1996-1944/14/5/1091/s1, Figure S1: Measured mass for 0.1 ml of (a) C-MH, (b) CTAB-DP-MH and (c) N-DP-MH samples.

## Nomenclature

I_001_, I_101_, I_110_	intensity reflections respectively of [001], [101] and [110] planes
m_in_	initial sample mass (g)
m_ins*t*_	instantaneous mass (g)
$M_{\mathrm{Mg}\left(\mathrm{OH}\right){\;}_{2}}$	molecular weight of Mg(OH)_2_ (g mol^−1^)
M_MgO_	molecular weight of MgO (g mol^−1^)
$Q_{s/r}^{M}$	stored/released heat capacity per mass unit ($\mathrm{kJ}/{\mathrm{kg}}_{\mathrm{Mg}{\left(\mathrm{OH}\right)}_{2}}$)
$Q_{s/r}^{V}$	stored/released heat capacity per volume unit (MJ m^−3^)
S_BET_	specific surface area (m^2^ g^−1^)
T_d_	dehydration temperature (°C)
T_h_	hydration temperature (°C)
V_PORE_	pore volume (cm^3^ g^−1^)
*Greek symbols*	
β	reacted fraction (%)
β ^f^_d_	reacted fraction at the end of the dehydration treatment (%)
β ^i^_d_	reacted fraction at the beginning of the dehydration treatment (%)
β_h_	final reacted fraction of MgO at the point of water supply termination (%)
Δm_rea*l*_	instantaneous real mass change (%)
Δm_th_	theoretical mass change due to the dehydration of Mg(OH)_2_ normalized to the total amount present in the sample (%)
Δβ_d_	dehydration conversion (%)
Δβ_h_	hydration conversion (%)
ΔH^0^	dehydration/hydration reaction enthalpy (kJ mol^−1^)
ρ	bulk density (kg m^−3^)
