# Supplementary material for: Tuning Mg(OH)2 Structural, Physical, and Morphological Characteristics for Its Optimal Behavior in a Thermochemical Heat-Storage Application
Source: Materials (Basel). 2021 Feb 26;14(5):1091. doi: 10.3390/ma14051091 (PMC7956584; doi:10.3390/ma14051091)
Supplement: Supplementary file 1 [file materials-14-01091-s001.pdf]

materials

MDPI

Supplementary Materials

Tuning $Mg(OH)_2$ Structural, Physical, and Morphological Characteristics for Its Optimal Behavior in a Thermochemical Heat-Storage Application

Elpida Piperopoulos [1,2,*], Marianna Fazio [1], Emanuela Mastronardo [3], Maurizio Lanza [4] and Candida Milone [1,2]

[1] Department of Engineering, University of Messina, 98166 Messina, Italy; faziom@unime.it (M.F.); cmilone@unime.it (C.M.)
[2] National Interuniversity Consortium of Materials Science and Technology (INSTM), 50121 Florence, Italy
[3] Institute of Catalysis and Petrochemistry, Spanish National Research Council (CSIC), E-28049 Madrid, Spain; e.mastronardo@csic.es
[4] Institute for Chemical and Physical Processes (IPCF)—CNR, 98158 Messina, Italy; lanza@ipcf.cnr.it
* Correspondence: epiperopoulos@unime.it

Citation: Piperopoulos, E.; Fazio, M.; Mastronardo, E.; Lanza, M.; Milone, C. Tuning $Mg(OH)_2$ Structural, Physical, and Morphological Characteristics for Its Optimal Behavior in a Thermochemical Heat-Storage Application. *Materials* 2021, *14*, 1091. https://doi.org/10.3390/ma14051091

Academic Editor: Simona Bennici

Received: 21 January 2021
Accepted: 23 February 2021
Published: 26 February 2021

Publisher's Note: MDPI stays neutral with regard to jurisdictional claims in published maps and institutional affiliations.

Copyright: © 2021 by the authors. Submitted for possible open access publication under the terms and conditions of the Creative Commons Attribution (CC BY) license (http://creativecommons.org/licenses/by/4.0/).

It is shown in Figure S1 the lower mass measured for the same powder volume (0.1 ml) of (a) C-MH sample with respect to (b) CTAB-DP-MH and (c) N-DP-MH samples.

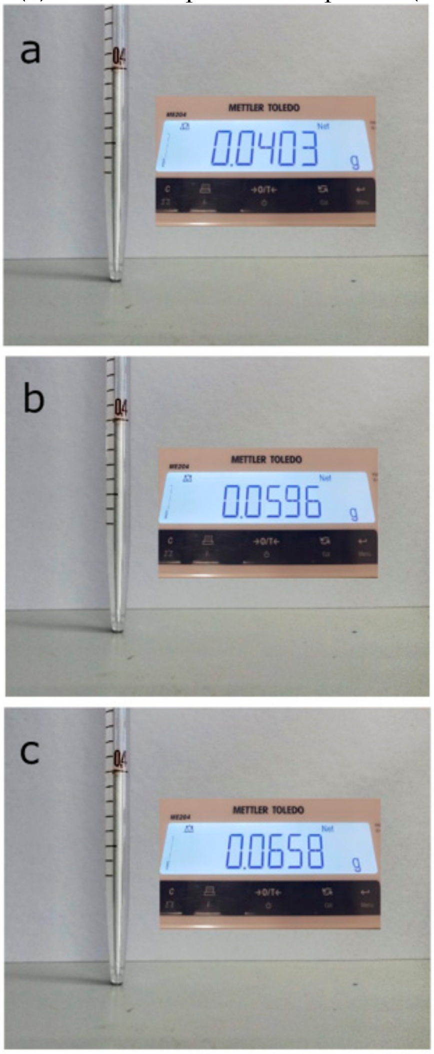

Figure S1. Measured mass for 0.1 ml of (**a**) C-MH, (**b**) CTAB-DP-MH and (**c**) N-DP-MH samples.
